# Some Diseases of the Foot*From notes of lectures on Pathological Shoeing delivered by Dr. E. A. MacLellan before the Columbia Veterinery College and School of Comparative Medicine.

**Published:** 1882-10

**Authors:** Henry C. Slee


### ART. V.—SOME DISEASES OF THE FOOT.*

BY HENRY C. SLEE.

KNUCKLING AT THE FETLOCK may arise from disease of the joint, from unskilful paring of the foot, or from excessive strain. Too great length of toe may bring an unnatural strain on the posterior tendons; or pulling too heavy loads may have the same effect, especially in young horses. In some cases the cause is obscure, corresponding apparently to the weakness of the ankle joint so common in scrofulous human beings—a natural weakness of the tendons—and as the great strain is brought to bear upon the flexors, when sufficient exciting cause is applied, a sub-acute inflammation is set up, an exudate is poured out on the tendon fibres, which diverts them from their naturally straight course, and consequently contracts them. The corresponding weakness in the anterior tendons allows them to relax, but not being subject to the same strain, inflammation does not attack them. The perforans and perforatus tendons only are involved—differing from the condition known as "knee-sprung," where the flexors of the metacarpus also are involved.

If the column of bones is distorted from its natural bearings by abnormal position of the foot, we may as a result have knuckling at the fetlock, or descent of the fetlock, or sprung knee—one or other of these conditions depending on the form of the limb. If the humerus is short, and thus greater strain put upon the knee, when this predisposition exists, the animal is apt to become "knee-sprung" instead of "knuckled." Contraction or tenderness of the heels, causing a flexed position for relief or inability to bring the foot fairly down, may cause contraction of the tendons, and if the knees be well formed may result in "knuckling;" or the inflammation originated by contraction of the heels may extend to the naturally weak tendons.

The treatment is only palliative. If attended to early, the disease may sometimes be easily averted until a recurrence of the exciting cause. The foot should be carefully put in proper shape,

* From notes of lectures on Pathological Shoeing delivered by Dr. E. A. MacLellan before the Columbia Veterinery College and School of Comparative Medicine.

the inflammation reduced by treatment according to its intensity in each case, chloroform liniment generally being best. In some cases, a high-heeled shoe, in others the lowering of the heels, is necessary to relieve the tendons of strain, and the joint will return to its normal condition. High toes sometimes do harm by increasing the inflammation. In cases of long standing, after properly adjusting the foot, blistering and even firing, succeeded by a period of rest at grass, often proves beneficial.

In cases which resist all other treatment tenotomy may be resorted to. The extent to which the tendons will be replaced is surprising. A gap of four inches has been known to be filled up in six weeeks.* The new tendon thus formed is generally thick and clumsy. Great care must be taken in adjusting the foot—bringing it so directly under the line of the leg that the pressure may be equalized.

In old cases the ligaments also become affected, so that the joint is hardly likely to be held in position long if straightened, and will never resist strain; but the form of the joint may be temporarily restored even in very bad cases by tenotomy.

### LAMINITIS.

The name simply implies inflammation of the laminæ, and is hardly comprehensive enough; for a diseased condition of both cartilage and bone frequently accompanies it. In its simplest form, however, it is an inflammation of the sensitive laminæ, with a tendency to destruction of the whole foot.

The predisposing causes are:

I.—Excessive fatness. Whether this affects the predisposition in any manner other than adding to the weight, seems improbable; but it is no doubt sometimes a predisposing cause.

II.—Bad shoeing. The laminæ are naturally placed in such a direction that they receive the pressure in the line of their greatest strength, and if, by raising the heel too high, etc., the weight is diverted from this line and exerted in another direction on them, inflammation is the result. Thinning of the sole is a common cause under this head. The wall, deprived of its support at the plantar surface, either contracts or expands, and the laminæ suffer in either case.

---

*Case: Dr. G. H. Berns, Brooklyn.

III.—Badly formed feet. Horses with too wide feet are generally subject to laminitis.

The exciting causes are:

I.—Concussion. The violent concussion, either in rapid driving or hard pulling over pavements, causes over-stimulation of the laminæ, and great determination of blood to them, and before this has had an opportunity to subside by gentle exercise, if the animal is placed in the stable, with the feet distended with blood, one of the great factors in the return of the blood to the heart (*i. e.*, exercise) is taken away, and inflammation is the result.

II.—Exhaustion. When, from any cause, the animal becomes exhausted, the weakest part is sure to suffer. Even while standing in the stable the laminæ have to sustain the weight, notwithstanding they may be exhausted with over-straining. Sometimes, when lame, one foot cannot sustain the weight, the other foot, having to do double duty, becomes exhausted, resulting in laminitis. The well-known tendency of sea voyages to produce laminitis can be traced to this cause, the constant effort to stand straight causing a strain on the laminæ, with consequent exhaustion.

III.—Rapid changes of temperature. If, while over-heated, the animal drinks cold water, the effect is to drive the blood to the surface and the extremities; exposing heated horses to cold wind, as may often be observed on ferry-boats, drives the blood from the surface, and with the same result of determination to the laminæ; and the laminæ, being already, perhaps, in a semi-diseased condition, from some of the predisposing causes, and possibly the feet being unable to relieve themselves by sweating, laminitis is the natural result.

Washing horses while hot (by the hose with cold water) is apt to set up a passive congestion which will develop laminitis very quickly if the predisposing causes have been at work.

IV.—Purgative medicines have sometimes induced this condition.

V.—Localization of fever in pneumonia, entiritis, etc. The feet are peculiarly liable to take on this disease from an irritable condition of the system, owing doubtless, to their position and anatomical construction.

The symptoms are usually developed in one night. In the morning the animal exhibits a disinclination to move; the pulse is full and bounding; respiration hurried; the feet hot and dry, and tapping them gently with a hammer evidently causes great pain; the animal will resist any effort made to raise the feet; will give evidence of great suffering, and will probably sweat. If the two fore feet are involved they will be advanced, and the weight thrown upon the heel, the hind feet brought well under the body so as to relieve the fore feet in sustaining the weight. If the hind feet are involved they will be thrown forward with the weight on the heel, and the fore feet well back. If all the feet are involved they will be lifted alternately with every evidence of pain. If an effort is made to move the animal it will not move its feet until its head is so far turned round that it moves apparently to regain its balance, and if started forward will probably stumble so as to almost strike its nose against the ground. If it has opportunity it will lie down on one side, with head and legs stretched out, and on being compelled to rise will evidently suffer terribly until the feet become accustomed to the weight. In walking the feet will be advanced and the heels brought to the ground first, and if the hind feet are unaffected they will be brought forward with a jerk to relieve the forward feet.

There may be simple congestion which will readily yield to treatment; or there may be active inflammation, which may terminate in resolution; or the sub-acute or chronic form of the disease may be developed—the latter usually depending for their continuance on structural changes. The exudation may be liquefied and absorbed, or may go on to suppuration; the inflammation may extend to the pedal bone, and periostitis, ostitis or necrosis result. If the disease does not yield to treatment within a week, the sub-acute form is generally developed, and with this come dropping of the sole, turning up of the toe, seedy toe or pumiced feet. These deformities of the toe are due to the exudate pressing between the horn and the bone, forcing the horn up and the bone down; the sensitive and horny laminæ are separated, and the tension on the flexor tendons forces the bone down; the nutrition being interfered with, an unequal growth of horn is the result; and the imperfectly formed horn from the sensitive laminæ constitutes "seedy toe." One cause of the turning up of the toe is the greater growth of horn at the posterior portion of the foot, where its growth is less interfered with, and as it forces itself forward and almost under the toe, the latter, not growing in proportion straight down from the coronet, is turned up. This condition may be aggravated by improper shoeing—throwing too much weight upon the toe. Atrophy of the antea-spinatus muscle often accompanies this condition.

Laminitis is a disease in which preventive treatment may and should always be adopted; and one of the principal things in this is good shoeing. If the foot is very flat and wide, clips should be placed on the outside of the shoe, and the shoe allowed to rest upon the sole if it can be borne. The animal should not be driven when his feet are overgrown or hard, nor should an animal from the country be overworked on the city pavements until accustomed to the change. When Western horses are brought here their feet gradually change in form—if rapidly, lameness results; but if slowly, only slight inflammation is induced, which, however, decidedly predisposes to laminitis. The feet should not be allowed to become too dry, and the animal should always be moved about until gradually cooled after being heated. When excessively lame on one foot, the well foot beside it, bearing the extra strain, should be carefully watched and not allowed to become inflamed. In many cases it is better to put the animal in a sling than to allow it to keep the additional weight on one foot for a long time; and wet cloths should be applied to keep-down incipient inflammation.

After the development of the disease the treatment must vary according to the intensity of the inflammation and the form of the feet. If the feet are thin-soled and flat the shoes had better be left on, but if comparatively strong the shoes may be removed, and the animal placed on a deep soft bed, the feet soaked in water and large poultices applied. This soaking and poulticing must be kept up and large doses of nitrate of potass administered internally. On the second day the following powder may be administered two or three times during the day:

Calomel, grains, xx.
Bismuth subnit., ʒ ii.
Morphia, grains, ii. to iii.

the amount of morphia being regulated by the intensity of the pain.

Great care must be observed in keeping up the anti-flogestic treatment incessantly until all danger has passed. In the very early stages sweating is sometimes of service, but not after the disease has fully set in.

After the inflammation has subsided the cause of the disease should be removed by proper shaping of the foot, etc.

In later and severer stages the feet should be soaked for an hour or more in hot water ; and after that, cold poultices applied. Aconite may be given if the fever is high, and the pain relieved by opium.

When the acute symptoms have subsided the feet should be soaked in cold water, and iodide of potassium administered internally.

In shoeing in the sub-acute or chronic form of this disease, the heel should be lowered, the shoe set well back from the toe, and as much sole-pressure allowed as the nature of the foot will admit of. Clips should be turned up on the outside of the shoe to prevent the foot spreading. In some cases a bar shoe should be applied and frog-pressure allowed. If, as is often the case, the sole is found worn away in front of the frog, so that it will yield on slight pressure, a leather sole may be applied, and the shoe nailed on over it.

